# Targeted next-generation sequencing for pediatric community-acquired pneumonia pathogen detection: a single-center study

**DOI:** 10.3389/fcimb.2026.1827409

**Published:** 2026-07-29

**Authors:** Luoman Yan, Haiyan Zhang, Hengheng Fu, Hao Dong, Junjie Chen, Lin Yu, Lei Zhang

**Affiliations:** 1 Chengdu Women’s and Children’s Central Hospital, School of Medicine, University of Electronic Science and Technology of China, Chengdu, China; 2 Department of pediatrics, The Affiliated Hospital of Southwest Medical University, Sichuan Clinical Research Center for Birth Defects, Luzhou, Sichuan, China

**Keywords:** bacterial culture, children, community-acquired pneumonia, targeted next-generation sequencing, virus detection

## Abstract

**Background:**

Community-acquired pneumonia (CAP) is a major contributor to the high burden of acute lower respiratory tract infections in children, characterized by significant incidence and mortality. While targeted next-generation sequencing (tNGS) provides a rapid, accurate, and comprehensive approach to pathogen identification, potentially enabling timely and precise clinical decisions, its application in pediatric populations is not well established. Therefore, this study was designed to investigate the clinical utility of tNGS for detecting pathogens in children with CAP.

**Methods:**

This retrospective study enrolled 838 children diagnosed with CAP, who were admitted to Chengdu Women and Children’s Central Hospital and underwent tNGS testing between February 2023 and May 2025. Additionally, the results of conventional microbiological tests were collected for comparative analysis to characterize the respiratory pathogen profile in this pediatric cohort.

**Results:**

The effective detection rate of tNGS (94.0% 788/838) was significantly higher than that of conventional microbiological tests (CMTs) (71.0% 483/680), and the combination of both methods increased the rate to 97.3% (815/838) (P < 0.0001). In contrast to its superior bacterial and atypical pathogen detection, tNGS showed a lower viral detection rate than CMTs. A high positive agreement between the two methods was observed for *Mycoplasma pneumoniae* (MP) and *Haemophilus influenzae*, whereas a high negative agreement was found for viral infections. MP was the most frequently detected pathogen overall, followed by *Haemophilus influenzae* and *Streptococcus pneumoniae* among bacteria, and rhinovirus and respiratory syncytial virus (RSV) among viruses. Age-specific analysis revealed distinct patterns: viral pathogens were more frequently detected in infants, who also showed the highest detection rate of *Staphylococcus aureus*; preschool children were prone to bacterial infections; and school-aged children were predominantly affected by atypical pathogens.

**Conclusion:**

Compared to CMTs, tNGS demonstrated superior capability in detecting a broader range of pathogens with higher sensitivity, making it particularly valuable for identifying coinfections. When interpreted alongside clinical manifestations, tNGS provides clinicians with a precise diagnostic tool for the early identification of lower respiratory tract pathogens. This information, when integrated with clinical presentation, can guide more timely and appropriate therapeutic decisions, which may help mitigate disease severity and reduce mortality in children with CAP.

## 1 Introduction

Lower respiratory tract infections represent a significant health burden in children under five, accounting for substantial morbidity and a mortality rate of 13.3% (GBD 2019 Under-5 Mortality Collaborators, 2021). Among these, community-acquired pneumonia (CAP) is a predominant contributor, caused by a wide spectrum of pathogens, including bacteria, viruses, and fungi (Torres et al., 2021; GBD 2019 LRI Collaborators, 2022). Accurate and timely identification of the causative agent is essential for effective clinical management. However, current conventional microbiological tests (CMTs) face critical limitations. Firstly, methods like bacterial culture and serology are time-consuming. Secondly, even molecular methods like qPCR have a restricted detection scope. These shortcomings delay accurate diagnosis, hinder optimal antibiotic stewardship, and may promote antimicrobial resistance, thereby increasing treatment complexity (Lin et al., 2025).

Currently, next-generation sequencing (NGS) technologies—both metagenomic (mNGS) and targeted (tNGS)—represent the forefront of accurate and rapid pathogen detection in respiratory specimens such as sputum and bronchoalveolar lavage fluid (BALF) (Wang et al., 2022). Although mNGS offers comprehensive pathogen coverage and is adept at detecting rare or fastidious organisms, its widespread application is hindered by several factors: high cost, difficulty differentiating colonization from infection, and the absence of standardized interpretation guidelines, especially given the complexity of the respiratory microbiome (Diao et al., 2022; Su et al., 2024). In contrast, tNGS, which integrates PCR with NGS, circumvents many of these drawbacks. Notably, studies have shown that tNGS achieves diagnostic performance comparable to mNGS for respiratory pathogens, may be superior for DNA virus detection, and provides this at a substantially lower cost while still covering up to 95% of respiratory infection etiologies (Li et al., 2022; Sun et al., 2024). Moreover, tNGS demonstrates high concordance with both clinical diagnoses and conventional test results, and its resistance gene detection correlates well with phenotypic antimicrobial susceptibility (Zhang et al., 2024). Nevertheless, evidence regarding the application of tNGS specifically for pediatric CAP remains scarce, and its clinical utility in this population requires further validation.

This retrospective study analyzed pathogen testing data from 838 children who underwent both tNGS and CMTs. We aimed to compare the detection rates of these two methods for pulmonary pathogens, evaluate the diagnostic utility of tNGS in pediatric CAP, and characterize the respiratory microbial profile in this cohort.

## 2 Materials and methods

### 2.1 Data source and ethical statement

We retrospectively analyzed data from 838 children hospitalized with CAP at Chengdu Women and Children’s Central Hospital from February 2023 to May 2025, all of whom underwent tNGS testing on BALF or sputum samples. Demographic information, clinical data, and results from conventional microbiological tests (including respiratory virus qPCR, sputum bacterial culture, and IgM antibody detection) were collected. Bronchoscopy was performed by experienced clinicians following safety protocols, with no serious adverse events reported. Samples were sent to a third-party laboratory for tNGS analysis. The study was approved by the Institutional Ethics Committee of Chengdu Women and Children’s Central Hospital and complied with the Declaration of Helsinki. Written informed consent for procedures was obtained from legal guardians; however, due to the retrospective and anonymized nature of the data analysis, the ethics committee waived the requirement for additional informed consent for this study.

### 2.2 Inclusion criteria

1. The diagnosis complied with the Chinese guidelines for the diagnosis and treatment of community-acquired pneumonia in children (2023 version); 2. During hospitalization, undergo tNGS testing or CMTs testing in BALF or sputum; 3. Age < 18 years old; 4. Child has complete demographic and clinical data, with no more than 20% of relevant data missing.

### 2.3 Exclusion criteria

1. There are congenital or secondary immune deficiencies, genetic metabolic diseases, severe hematological diseases and other diseases; 2. Have a history of taking immunomodulators; 3. Incomplete medical history information; 4. Refuse to conduct tNGS and CMTs tests.

### 2.4 Criteria for judging SCAP

Severe community-acquired pneumonia (SCAP) in children was defined as CAP presenting with any of the following: (1) severe respiratory distress (e.g., shortness of breath, persistent hypoxemia); (2) systemic toxic symptoms (e.g., persistent high fever, consciousness disorder); (3) major complications (e.g., heart failure, shock); or (4) radiographic findings of extensive pulmonary involvement, including large-area consolidation in one or both lobes, atelectasis, or moderate-to-large pleural effusion.

### 2.5 tNGS detection

BALF or sputum samples were collected for tNGS testing; For infants unable to expectorate, samples were obtained via nasopharyngeal suction. All samples were sent to a third-party laboratory (Chengdu Huayin Medical Laboratory) for tNGS analysis.

#### 2.5.1 Sample processing and nucleic acid extraction

Upon receipt, 600 µL of each sample was lysed using glass beads (0.5 mm) in a TissueLyser II (Qiagen, Hilden, Germany) at 30 Hz for 10 minutes. DNA and RNA were co-extracted using the TIANGEN Nucleic Acid Extraction Kit (TIANGEN Biotech, Beijing, China) according to the manufacturer’s instructions. Extracted nucleic acids were quantified by Qubit 4.0 (Thermo Fisher Scientific, Waltham, MA, USA) and stored at -80 °C until use.

#### 2.5.2 Library preparation and ultra-multiplex PCR

A targeted ultra-multiplex PCR assay was designed to simultaneously amplify 125 clinically common respiratory pathogens (including 68 bacteria, 42 viruses, 11 fungi, and 4 atypical pathogens). The primer pool was designed to target highly conserved regions of each pathogen’s genome, with amplicon sizes ranging from 150 to 300 bp. Multiplex PCR was performed using the Multiplex PCR Kit (Qiagen) with 20 ng of input DNA/cDNA under the following conditions: 95 °C for 15 min; 35 cycles of 94 °C for 30 s, 58 °C for 90 s, and 72 °C for 60 s; and a final extension at 72 °C for 10 min. PCR products were purified using AMPure XP beads (Beckman Coulter, Brea, CA, USA).

#### 2.5.3 Sequencing and bioinformatics

Libraries were sequenced on an Illumina MiniSeq platform (Illumina, San Diego, CA, USA) using 2 × 150 bp paired-end reads. A minimum of 2 million raw reads per sample was required. Raw sequencing data were demultiplexed and adapter-trimmed using fastp (v0.23.2). Human host sequences were removed by alignment to the human reference genome hg38 (GRCh38.p14) using bowtie2 (v2.4.5). Remaining reads were aligned to a curated reference database (RefSeq, NCBI, April 2024 release) containing complete genomes of the 125 target pathogens. Taxonomic classification was performed using Kraken2 (v2.1.2) with a confidence threshold of 0.7. Pathogen reads were normalized to reads per million (RPM) after host subtraction.

#### 2.5.4 Limit of detection and reporting threshold

The analytical limit of detection (LoD) of the assay, determined using quantified control plasmids spiked into negative BALF matrix, was 50 copies/mL for bacterial targets and 200 copies/mL for viral targets. However, to distinguish true infection from colonization or background contamination, a clinical reporting threshold of >1×10³ copies/mL was applied. This threshold was empirically established based on preliminary data from 50 pilot pediatric CAP samples and consensus among three senior clinicians (see section 2.7 for clinical adjudication process).

### 2.6 CMTs

Conventional microbiological tests included qPCR on nasopharyngeal swabs (for RSV, MP, *adenovirus, human rhinovirus, influenza A virus*, and *influenza B virus*), sputum culture, and serological testing. Sputum samples were collected prior to antibiotic administration—by expectoration into sterile tubes for older children or via nasopharyngeal suction for infants and toddlers, and cultured for 72 hours. Sputum culture was attempted in all patients but was successful (produced adequate specimen) in 680 cases, and 18 sputum samples were specifically submitted for tNGS.

Serum samples from a subset of patients were tested for IgM antibodies against common respiratory pathogens (*Legionella pneumophila*, MP, *Coxiella burnetii*, *C. pneumoniae*, *adenovirus*, RSV, *influenza A* and *B viruses*, and *parainfluenza virus*).

### 2.7 tNGS result determination

Due to the current lack of standardized interpretation criteria for tNGS, a stringent criterion of >1×10³ copies/mL was established to distinguish true pathogens from colonizing flora, thereby mitigating the influence of sequencing depth and background microbial load. All tNGS-positive results were then independently evaluated by two experienced pediatricians, who integrated clinical manifestations, conventional test results, and radiographic findings according to pediatric CAP guidelines. Any diagnostic discrepancy was resolved by consultation with a third senior clinician, ensuring the reliability of the final pathogen assignment.

## 3 Statistical methods

Data analysis was conducted using SPSS27.0 software, and P< 0.05 was considered statistically significant. Categorical data were expressed as frequency counts and percentages. Measurement data were expressed as mean ± standard deviation (x ± SD). The significance of categorical data was tested by chi-square test or Fisher’s exact test, and the consistency of the two detection methods was evaluated by Kappa coefficient (Tooth and Ottenbacher, 2004). Due to multiple comparisons, Bonferroni correction was applied where appropriate. Adjusted significance thresholds are noted in the relevant tables.

## 4 Result

### 4.1 Research population

A total of 838 children diagnosed with CAP and meeting the inclusion criteria were enrolled in this study. All patients underwent tNGS testing on lower respiratory tract specimens, including 820 BALF samples and 18 sputum samples. The cohort consisted of 436 males and 402 females, with a male-to-female ratio of 1.08:1. The mean age was 5.27 ± 3.36 years. Age distribution was as follows: 104 infants (< 1 year), 101 toddlers (1–3 years), 264 preschool children (3–6 years), and 369 school-aged children (> 6 years). Based on disease severity, 635 patients were classified as having SCAP and 203 as having non-severe CAP.

The number of patients who underwent each conventional test varied due to sample availability and clinical indications: qPCR was performed in 752 patients, sputum culture was attempted in all patients but yielded adequate specimens in 680 patients, and IgM antibody testing was performed in a subset of 19 patients based on clinical suspicion.

### 4.2 tNGS test results

**Figure 1:**
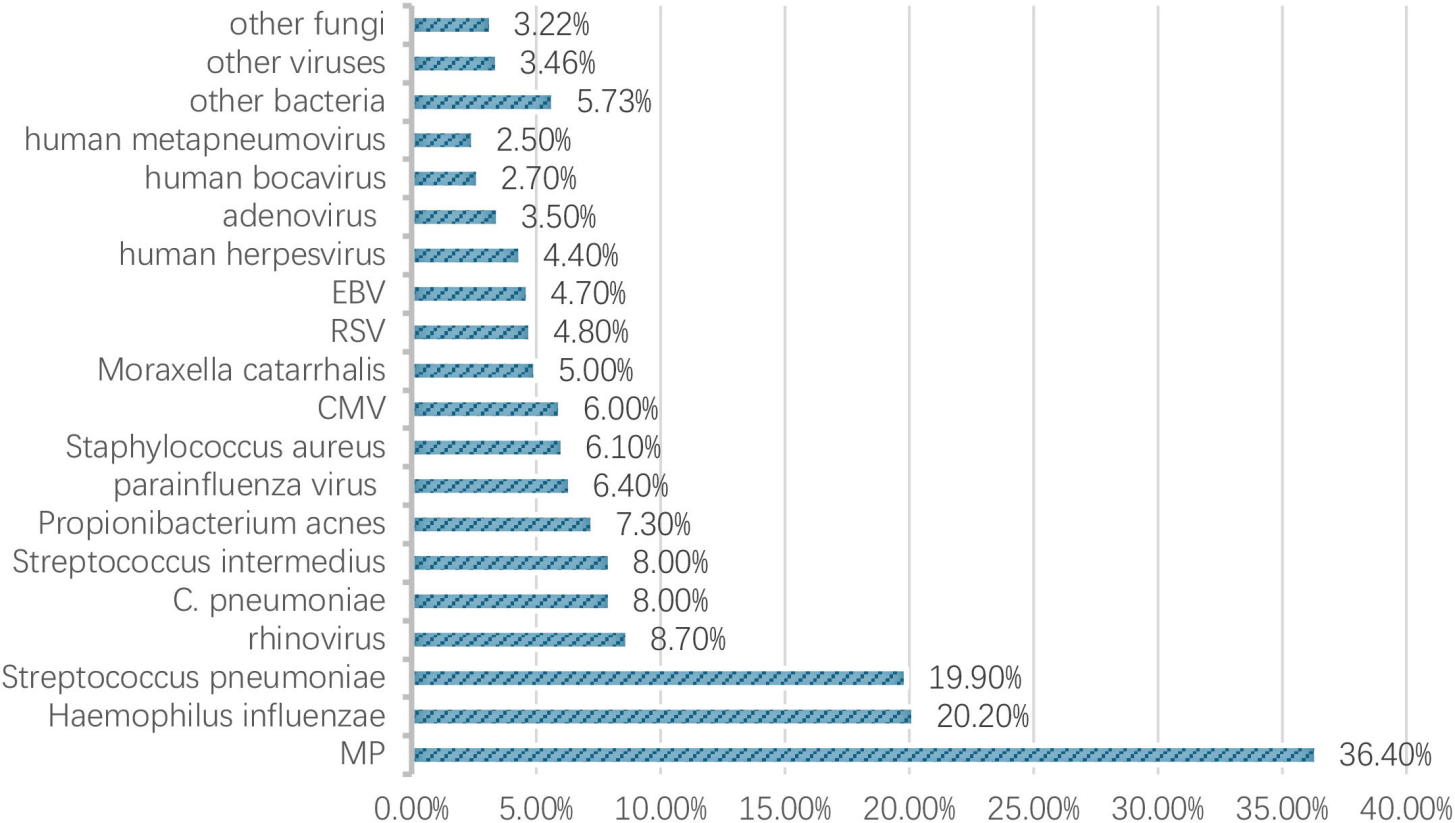
tNGS detection results of respiratory tract microorganisms in children with CAP.

tNGS demonstrated a total detection rate of 99.9% (837/838) and an effective detection rate of 94.0% (788/838) in this pediatric cohort. Total detection rate (99.9%) refers to any tNGS signal above the analytical limit of detection (50 copies/mL). Effective detection rate (94.0%) required the clinical reporting threshold (>1×10³ copies/mL) and independent clinical adjudication to exclude likely colonization or contamination. From the 788 effective cases, 42 pathogens were identified, including 20 bacteria, 16 viruses, 4 fungi, and 2 atypical pathogens (MP and *C. pneumoniae*). The most prevalent pathogen was MP (36.4%, 305/838), followed by *Haemophilus influenzae* (20.2%, 169/838), *Streptococcus pneumoniae* (19.9%, 167/838), *rhinovirus* (8.7%, 73/838), *C. pneumoniae* (8.0%, 67/838), and *Streptococcus intermedius* (8.0%, 67/838). Other detected organisms with frequencies ≥3% included *Propionibacterium acnes* (7.3%), *parainfluenza virus* (6.4%), *Staphylococcus aureus* (6.1%), *cytomegalovirus* (CMV) (6.0%), *Moraxella catarrhalis* (5.0%), RSV (4.8%), *Epstein-Barr virus* (EBV) (4.7%), *human herpesvirus* (4.4%), and *adenovirus* (3.5%). The complete pathogen profile, including less frequently detected microorganisms, is presented in **Figure 1**.

### 4.3 CMTs results

**Figure 2:**
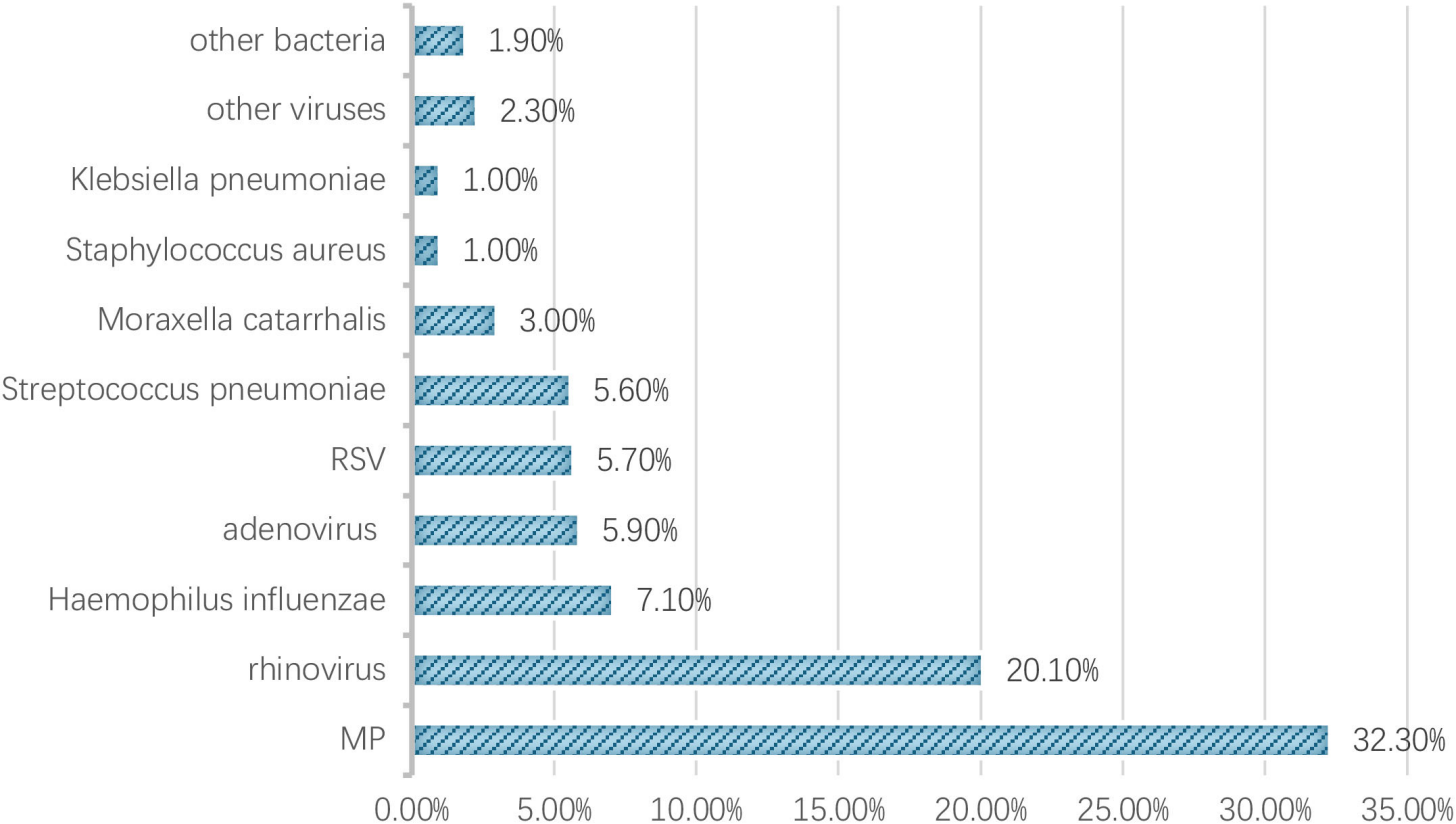
CMTs results of respiratory microbiota in children with CAP. Identical letters indicate no significant difference (p > 0.05), whereas different letters indicate a significant difference (p < 0.05).

**Figure 3:**
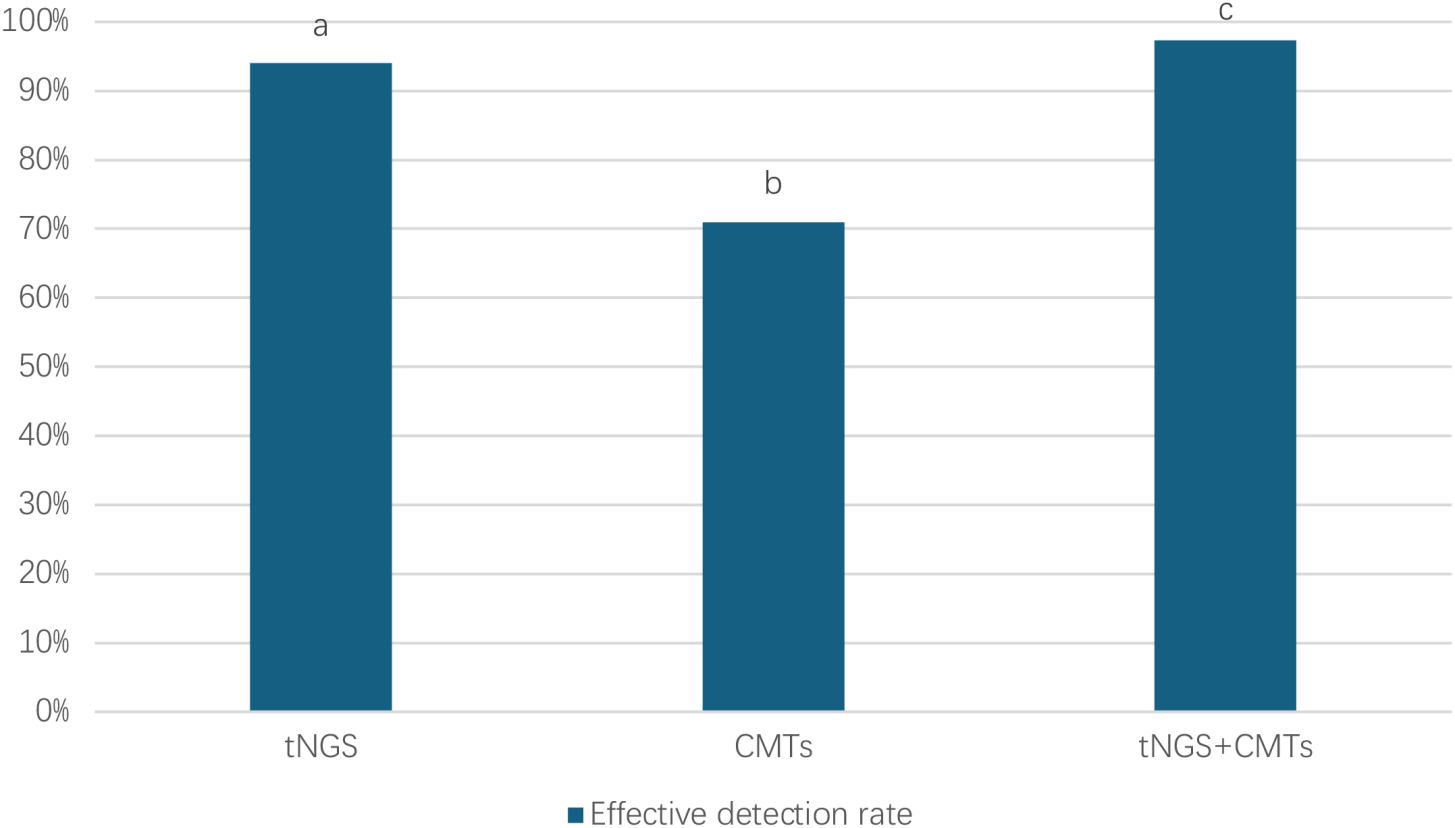
Comparison of effective detection rates of different detection methods for respiratory microorganisms in children with CAP. Identical letters indicate no significant difference (p > 0.05), whereas different letters indicate a significant difference (p < 0.05).

CMTs achieved an overall effective detection rate of 71.0% (483/680), with method-specific rates as follows: qPCR 58.9% (443/752), sputum culture 19.6% (133/680), and IgM antibody testing 47.4% (9/19). A total of 18 pathogens were identified by CMTs, including 11 bacteria, 6 viruses, and MP. MP was the most frequently detected pathogen (32.3%, 243/752), followed by *rhinovirus* (20.1%, 151/752), *Haemophilus influenzae* (7.1%, 48/680), *Streptococcus pneumoniae* (5.6%, 38/680), *adenovirus* (5.9%, 44/752), and RSV (5.7%, 43/752) (**Figure 2**). When combined, tNGS and CMTs increased the overall detection rate to 97.3% (815/838). Importantly, the effective detection rate of tNGS (94.0%, 788/838) was significantly higher than that of CMTs (71.0%, 483/680) (χ² = 293.141, P = 2.2141E-64) (**Figure 3**).

### 4.4 Consistency comparison between tNGS and CMTs

Using qPCR and sputum culture results as reference standards, we calculated the positive and negative percent agreement between tNGS and CMTs for eight common respiratory pathogens. Among these, MP demonstrated the highest positive agreement, followed by *Haemophilus influenzae* and *Streptococcus pneumoniae*. In contrast, positive agreement for viral pathogens was generally low. The detailed concordance between tNGS and CMTs detection is presented in **Table 1**.

**Table 1:** Consistency comparison of tNGS and CMTs detection for common respiratory pathogens in children.

Pathogens	Positive percent agreement	Negative percent agreement	Kappa
MP	89.30% (217/243)	87.43% (445/509)	0.737
*rhinovirus*	27.15% (41/151)	96.01% (577/601)	0.294
*Haemophilus influenza*	64.58% (31/48)	83.07% (525/632)	0.255
*adenovirus*	45.45% (20/44)	98.87% (700/708)	0.534
RSV	48.84% (21/43)	98.31% (697/709)	0.529
*Streptococcus pneumoniae*	50% (19/38)	82.71% (531/642)	0.153
*Moraxella catarrhalis*	35% (7/20)	96.21% (635/660)	0.242
*Influenza A virus*	28.57% (4/14)	99.73% (736/738)	0.393

Identical letters indicate no significant difference (p > 0.05), whereas different letters indicate a significant difference (p < 0.05).

Kappa coefficients were interpreted as: <0.00 poor, 0.00–0.20 slight, 0.21–0.40 fair, 0.41–0.60 moderate, 0.61–0.80 substantial, 0.81–1.00 almost perfect. Accordingly, MP showed substantial agreement (κ=0.737), adenovirus and RSV moderate agreement (κ=0.534 and 0.529), and other pathogens fair to slight agreement.

### 4.5 Pathogen co-occurrence analysis

To explore the frequency of pathogen co-detection, we constructed a co-occurrence heatmap for the eleven most prevalent pathogens (**Supplementary Figure 1**). Among the 788 tNGS-positive cases, 412 (52.28%) had a single pathogen detected, 224 (28.43%) had two pathogens, and 152 (19.29%) had three or more. The most frequent co-occurrence pair was *Streptococcus pneumoniae* with *Haemophilus influenza* (n=60), followed by *Haemophilus influenza* with MP (n=33).

### 4.6 Epidemiological characteristics of respiratory pathogens

**Figure 4:**
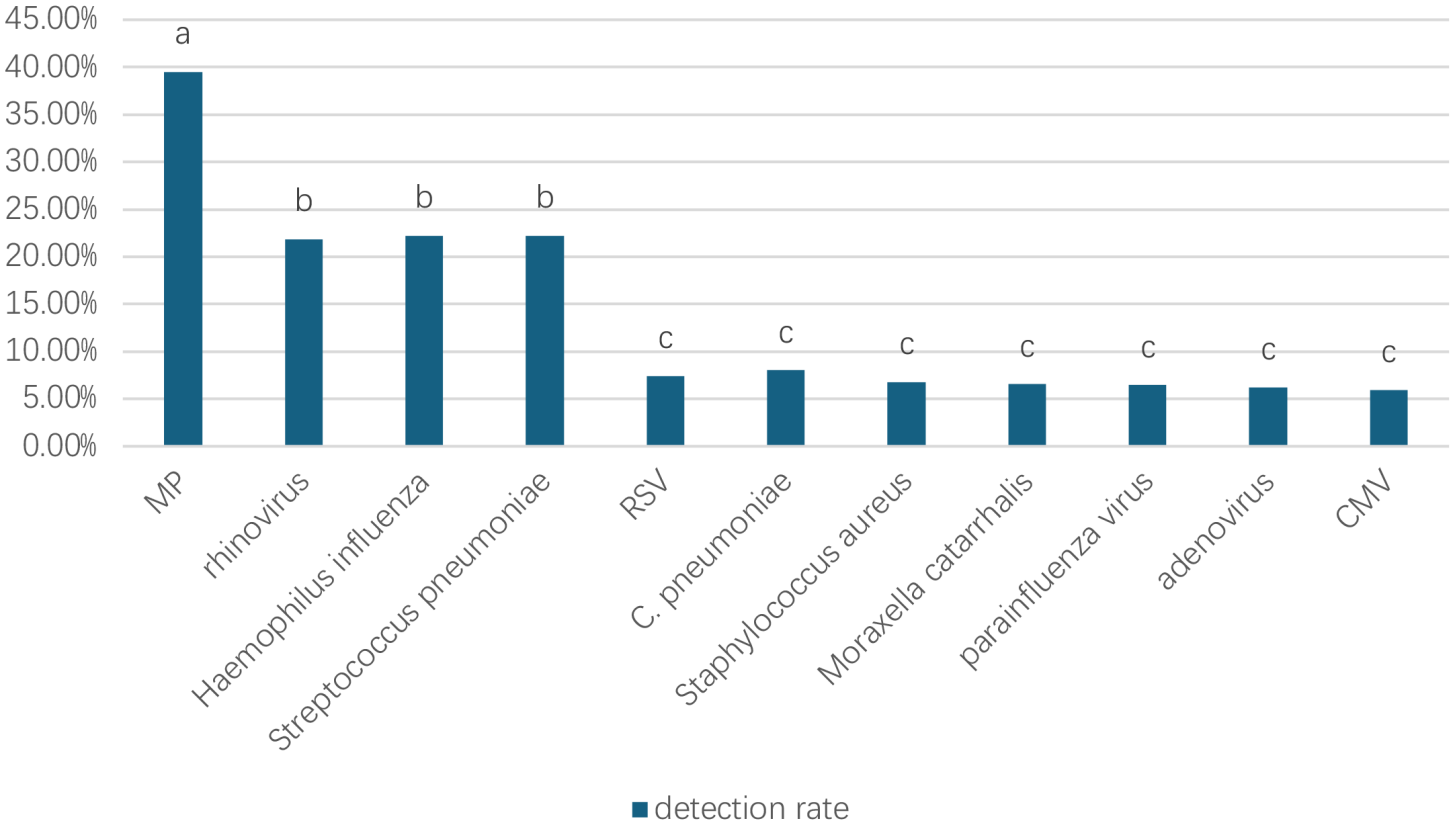
Comparison of the total detection rates of common respiratory pathogens in children with CAP. Identical letters indicate no significant difference (p > 0.05), whereas different letters indicate a significant difference (p < 0.05).

Epidemiological analysis was performed for 11 common respiratory microorganisms, examining their overall detection rates, as well as age and gender susceptibility patterns. As shown in **Figure 4**, MP exhibited the highest total detection rate at 39.50% (331/838), followed by *Haemophilus influenzae* and *Streptococcus pneumoniae*, both at 22.20% (186/838). *Rhinovirus* ranked next with a detection rate of 21.84% (183/838). Other detected pathogens included *C. pneumoniae* (8.00%, 67/838), RSV (7.40%, 62/838), *Staphylococcus aureus* (6.80%, 57/838), *Moraxella catarrhalis* (6.56%, 55/838), *parainfluenza virus* (6.44%, 54/838), *adenovirus* (6.21%, 52/838), and CMV (5.97%, 50/838).

Analysis of demographic susceptibility patterns revealed that male children were more susceptible to CMV (x^2^ = 4.165, P = 0.041) and *Streptococcus pneumoniae* (x^2^ = 4.139, P = 0.042), with no significant gender differences for other pathogens. But after Bonferroni correction for 11 comparisons (adjusted P = 0.0045), the nominally significant differences observed for CMV and *Streptococcus pneumoniae* were no longer statistically significant. Age-stratified analysis showed distinct profiles: school-aged children were predominantly affected by atypical pathogens (MP and *C. pneumoniae*); preschool children by *Haemophilus influenzae* and *Moraxella catarrhalis*; and infants and toddlers by RSV, *parainfluenza virus*, and CMV. Notably, after Bonferroni correction for 11 comparisons*, adenovirus, rhinovirus and Staphylococcus aureus* showed no significant differences across age groups (**Table 2**).

**Table 2:** Age distribution of different respiratory pathogens in children with CAP.

Pathogens	Infant positivity rate	Toddler positivity rate	Preschool children positivity rate	School-age children positivity rate	X2	P value (Bonferroni correction)
MP	11.54% (12/104)	23.76% (24/101)	36.74% (97/264)	53.66% (198/369)	76.287	1.9193E-16
*Streptococcus pneumoniae*	17.31% (18/104)	32.67% (33/101)	27.27% (72/264)	17.07% (63/369)	17.407	0.000583
*Haemophilus influenza*	25.00% (26/104)	21.78% (22/101)	29.92% (79/264)	15.99% (59/369)	17.846	0.000473
*Moraxella catarrhalis*	8.65% (9/104)	8.91% (9/101)	9.85% (26/264)	2.98% (11/369)	14.017	0.002883
*C. pneumoniae*	0.96% (1/104)	1.98% (2/101)	3.41% (9/264)	14.91% (55/369)	43.462	1.9633E-9
RSV	18.27% (19/104)	12.87% (13/101)	6.44% (17/264)	3.52% (13/369)	30.798	9.3767E-7
*Parainfluenza virus*	17.31% (18/104)	12.87% (13/101)	4.17% (11/264)	3.25% (12/369)	35.788	8.3042E-8
*adenovirus*	3.85% (4/104)	11.88% (12/101)	7.20% (19/264)	4.61% (17/369)	8.651	0.034316
CMV	29.81% (31/104)	4.95% (5/101)	3.03% (8/264)	1.63% (6/369)	121.994	2.87E-26
*Staphylococcus aureus*	12.50% (13/104)	9.90% (10/101)	5.68% (15/264)	5.15% (19/369)	8.970	0.029698
*rhinovirus*	28.85% (30/104)	26.73% (27/101)	22.73% (60/264)	17.89% (66/369)	7.909	0.047940

Bonferroni correction was applied for the 11 age-group comparisons (one per pathogen). Statistical significance was defined as P < 0.05/11 = 0.0045. P-values below 0.0045 are considered significant after correction.

## 5 Discussion

CAP remains a leading cause of morbidity and mortality in children worldwide, underscoring the critical need for rapid and accurate pathogen identification to guide timely clinical interventions and improve patient outcomes (McAllister et al., 2019). In this study, we evaluated the diagnostic performance of tNGS for respiratory pathogen detection in pediatric CAP and compared it with CMTs. Our findings demonstrate that tNGS achieved a significantly higher detection rate than CMTs (P < 0.05), consistent with previous reports (He et al., 2025). Notably, MP emerged as the predominant pathogen in both detection platforms, with detection rates exceeding 30%. Furthermore, the positive and negative percent agreement between tNGS and CMTs for MP detection reached approximately 80%, a finding that diverges from some earlier studies (Xu et al., 2011). This discrepancy may be attributed to the global MP pandemic observed since 2023, which has been particularly pronounced in East Asia. In China, MP infection rates have surged to as high as 47.89% (Chen et al., 2024). The resultant high pathogen load in infected children likely minimizes methodological variability, potentially explaining the comparable detection performance between tNGS and CMTs observed in our cohort.

However, for other common pathogens, detection rates varied significantly between methods. Notably, CMTs demonstrated superior detection rates for respiratory viruses compared to tNGS, with tNGS showing positive agreement below 50% but negative agreement exceeding 96% for viral pathogens. This indicates that CMTs remain the preferred method for rapid viral detection in clinical settings. Nevertheless, tNGS proved valuable for detecting viruses in CMTs-negative samples, thereby increasing overall viral detection rates and reducing false negatives. Consistent with previous reports (Gu et al., 2025), we found that tNGS-assisted detection achieved a positivity rate of up to 81.1% in CMTs-negative samples. This high false-negative rate with CMTs may be attributed to sampling timing relative to infection dynamics. Studies on SARS-CoV-2 have demonstrated that viral load peaks in the upper respiratory tract during early symptomatic phases but declines more rapidly in the upper tract than in the lower tract as infection progresses, particularly in severe cases (Wölfel et al., 2020; Chen et al., 2021). Since qPCR typically uses nasopharyngeal swabs, CMTs are most effective during early infection. While CMTs offer advantages in turnaround time and cost, their limited sensitivity for low viral loads and mixed infections renders them suitable primarily for rapid diagnosis of common respiratory viruses in appropriate clinical contexts (Falzone et al., 2020). For patients with negative CMTs results but persistent high fever, suspected severe disease, or concern for atypical viral infections, tNGS should be considered as a complementary tool to enhance viral detection. Indeed, our study demonstrated that combining CMTs with tNGS increased the overall effective detection rate to 97.3%. From a logistical perspective, the turnaround time for tNGS in our study was approximately 48–72 hours from sample receipt to result report, which is longer than qPCR (4–6 hours) but comparable to bacterial culture (48–72 hours). The cost per tNGS sample was approximately 80 USD, which is higher than individual qPCR assays (15–25 per target) but substantially lower than mNGS (300–500 USD). Therefore, while tNGS may not replace qPCR for rapid point-of-care testing, it offers a cost-effective solution when a broad differential diagnosis or co-infection assessment is needed.

tNGS demonstrated superior bacterial detection compared to CMTs, particularly for *Haemophilus influenzae* (positive agreement 64.58%, negative agreement 83.07%) and *Streptococcus pneumoniae* (50% and 82.71%, respectively), indicating its ability to identify pathogens missed by sputum culture. While tNGS offers advantages in turnaround time, detection breadth, and identification of mixed infections—including fastidious organisms like *Mycobacterium tuberculosis* (Jin et al., 2025; Shang et al., 2025)—results must be interpreted cautiously due to potential false positives from background contamination or colonization (Shang et al., 2025). In our study, when tNGS and CMTs results disagreed, we did not automatically consider tNGS as correct. Instead, we used a clinical adjudication panel that reviewed each case’s complete clinical presentation, radiographic findings, and treatment response. For example, a tNGS-positive but CMTs-negative result for *Streptococcus pneumoniae* was classified as a true positive if the child had lobar consolidation and responded definitively to beta-lactam therapy; if the child was afebrile, had no radiographic infiltrate, and recovered without antibiotics, the tNGS result was considered likely colonization or contamination. This approach minimized misclassification bias but also highlighted the absence of a true gold standard—a limitation we address below.

Beyond pathogen detection, tNGS revealed respiratory microbial community characteristics, with *Streptococcus intermedius* and *Propionibacterium* species contributing to oral microbial colonization in early childhood (Kennedy et al., 2019). In the post-pandemic era, MP remains the predominant pathogen, followed by *H. influenzae*, *S. pneumoniae*, rhinovirus, and RSV, consistent with previous reports (Liu et al., 2023). MP exhibits 3–7 years epidemic cycles, influencing detection rates (Lee et al., 2021). Age-stratified analysis revealed distinct susceptibility patterns: infants and toddlers were more susceptible to viral infections; preschool children to bacterial infections; and school-aged children to atypical pathogens. These patterns reflect age-dependent factors including immunological maturation, anatomical development, and social exposure patterns (Handelman and Osborne, 1976; Liu et al., 2016; Deshmukh et al., 2025). In our study, infants showed the highest CMV detection rates among all age groups. This pattern is consistent with established understanding that postnatal CMV acquisition in infancy occurs primarily through breast milk from seropositive mothers, a transmission route well documented in the literature (Johnson et al., 2024). All CMV-infected infants were asymptomatic with normal hearing, consistent with previous studies (Lanzieri et al., 2017).

This study has several limitations. First, as a single-center study conducted in Chengdu, the findings may primarily reflect the local epidemiological characteristics of respiratory pathogens and may not be generalizable to the broader pediatric population. Second, the absence of a true gold standard for respiratory pathogen detection precluded the calculation of accurate sensitivity and specificity for either tNGS or CMTs; therefore, we could only assess the performance of tNGS through concordance analysis with conventional methods. Future studies addressing this limitation could employ latent class models or Bayesian methods to estimate sensitivity and specificity in the absence of a gold standard (Hui and Zhou, 1998; Dendukuri and Joseph, 2001). Third, the lack of standardized criteria for tNGS result interpretation may have led to underestimation of its true diagnostic performance, particularly for low-abundance microorganisms or those not detected by CMTs. Fourth, fungal pathogens were not included in the CMTs detection panel, preventing direct comparison of the two methods for fungal detection. Consequently, our findings should be interpreted with caution.

Future multicenter studies should address the following: (1) standardize tNGS interpretation criteria by establishing pathogen-specific quantitative thresholds based on bronchoalveolar lavage fluid from healthy controls; (2) incorporate longitudinal follow-up (e.g., 30-day outcomes) to assess the impact of tNGS results on antibiotic de-escalation, length of hospital stay, and mortality; (3) compare tNGS-guided therapy versus CMTs-guided therapy in a randomized controlled design. Such studies might reveal whether routine tNGS use reduces inappropriate antibiotic use by 20-30% and shortens hospitalization by 2–3 days in children with severe CAP.

## 6 Conclusion

In summary, while tNGS demonstrates superior diagnostic performance compared to CMTs for pediatric CAP, reliance on a single testing modality is insufficient for accurate etiological diagnosis. The optimal diagnostic strategy involves a rational selection of detection methods—guided by the stage of illness, clinical characteristics, and initial auxiliary findings—to effectively identify viral, bacterial, and atypical pathogens. Integrating tNGS results with clinical manifestations significantly enhances diagnostic accuracy. Notably, for children with SCAP, the combined use of CMTs and tNGS markedly improves pathogen detection, enabling timely therapeutic adjustments that can improve prognosis and reduce mortality.

## Data Availability

The raw data supporting the conclusions of this article will be made available by the authors, without undue reservation.
