# Supplementary material for: Targeted next-generation sequencing for pediatric community-acquired pneumonia pathogen detection: a single-center study
Source: Front Cell Infect Microbiol. 2026 Jul 29;16:1827409. doi: 10.3389/fcimb.2026.1827409 (PMC13461630; doi:10.3389/fcimb.2026.1827409)
Supplement: Supplementary file 1 [file DataSheet1.pdf]

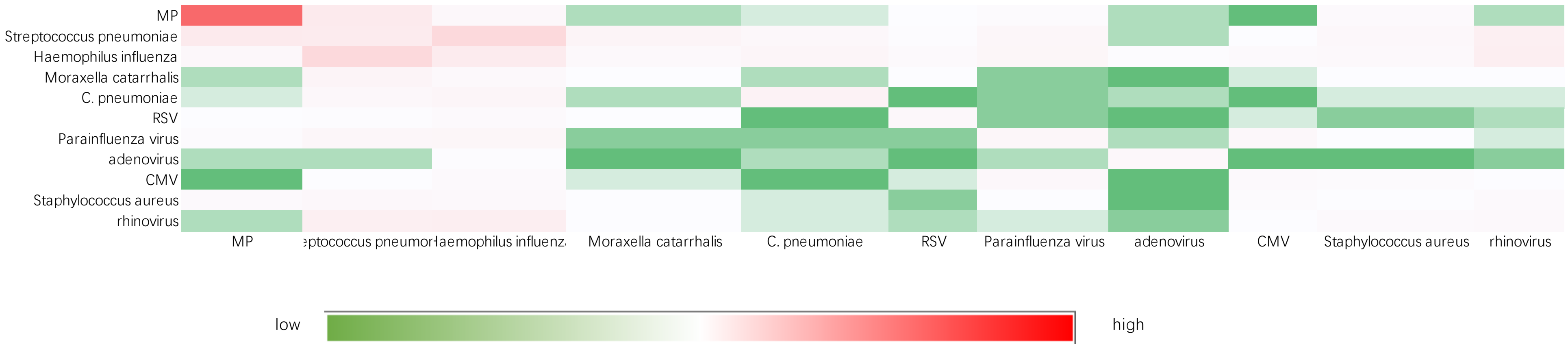
MP
Streptococcus pneumoniae
Haemophilus influenza
Moraxella catarrhalis
C. pneumoniae
RSV
Parainfluenza virus
adenovirus
CMV
Staphylococcus aureus
rhinovirus
MP
eptococcus pneumon
Haemophilus influenz
Moraxella catarrhalis
C. pneumoniae
RSV
Parainfluenza virus
adenovirus
CMV
Staphylococcus aureus
rhinovirus
low
high
